# An Account of the Effects of Laurel Water as Observed in the Bodies of Two Persons Who Died at Turin, January 22, 1785
*Extracted from the Memoirs of the Royal Academy of Sciences at Turin, for the years 1786 and 1787. 4to. Turin, 1788.


**Published:** 1790

**Authors:** M. Penchienati

**Affiliations:** Member of the Royal Academy of Sciences at Turin


VII. *An Account* * *of the Effects of Laurel Water as obſerved in the Bodies of two Perſons who died at Turin, January* 22, 1785. *By M.* Penchienati, *Member of the Royal Academy of Sciences at Turin.*

THE Lauro-Cerafus, ſo called by botaniſts on account of the reſemblance there is between its fruit and our cherries, was brought

* Extracted from the Memoirs of the Royal Academy of Sciences at Turin, for the years 1786 and 1787. 4to. Turin, 1788.

from from Trebizond into France in 1576. Its pernicious effects, however, had been known long before to the ancient Greeks and Romans. Strabo, in his time, had obferved that animals who tafted it died foaming at the mouth, like thofe affected with epilepfy. *Erat arbor*, fays his Tranflator, *lauro fimilis, qua guftata, jumenta omnia cum fpuma moriebantur, in morem comitialis morbi.* Apuleius, in the fourth book of his "Golden Afs," affures us that the fruit of this plant is hurtful to animals of every fpecies. Pliny afferts, that it is poifonous to goats, fheep, and horfes, and that its leaves are fo baneful to quadrupeds in general, that water in which thefe leaves have putrefied proves certainly fatal to the animals that drink it. The fame writer adds, that the juice of thefe leaves is a remedy of wonderful efficacy againft the bite of ferpents. Modern obfervations ferve to prove not only that this plant is poifonous to animals, but likewife that its diftilled water proves fuddenly fatal to man.

Notwithftanding the poifonous properties of this water, however, it feems that it has long been a practice to give five or fix drops of it, occafionally, to dogs as a ftomachic and means of fattening them; and in the Englifh, Irifh, and Italian

Cookery it is ufual to add a few drops of it to creams and cuftards, and likewife to brandy, for the fake of giving them a tafte like that of bitter almonds. This was probably done for a long time before the fatal effects of it were fufficiently ftriking to excite attention. The firft accounts we have of the death of any perfons from drinking of this water are thofe by Dr. Madden, phyfician at Dublin, inferted in the Philofophical Tranfactions for the year 1728. Thefe foon led to farther inquiries on the fubject among the Englifh, French, and Italian phyficians, and gave rife to a great number of experiments, from which it very clearly appeared that the diftilled water and oil of this plant are, in a certain dofe, a fatal poifon to animals in general. The Abbé Rofier, however, has related an inftance of a horfe who did not die, though he fwallowed feveral ounces of this water*.

It appears, then, from what I have faid, to be a fact long eftablifhed, that the lauro-cerafus is a poifon to animals, and that the water diftilled from it is not lefs fatal to man. But no perfon has hitherto defcribed the effects pro-

* See Vol. I. of this Journal, page 334.—Editor.

duced duced by it in the viſcera of thoſe who have had the misfortune to take it. Two fatal inſtances of this ſort, which occurred in this capital the 22d of January, 1785, have afforded me an opportunity of noticing theſe effects. The caſes themſelves are ſo extraordinary, that I have thought it right to deſcribe them ſomewhat minutely.

A man and a woman, in the ſervice of a nobleman in this city, having by miſtake taken up a bottle of laurel water, which was ſtanding among ſome cordials intended for the table, they each of them unfortunately ſwallowed about two ſmall ſpoonfuls of it, a doſe that proved ſufficient to occaſion inſtant death.

The dead bodies were immediately removed, by order of the Police, to the room adjoining to the Anatomical Theatre in the Univerſity, and were there examined by my colleague, M. Baldi, and myſelf, in the preſence of ſeveral phyſicians and ſurgeons.

We began with obſerving the external appearances of the two bodies, and remarked that in both the ſkin was of its natural colour. The eyelids were half open, and the pupil of the woman's left eye was more dilated than that of the right, and even præternaturally ſo. In both ſubjects the mouth was ſtrongly cloſed, with a ſlight appearance of a frothy moiſture between the lips, ſo that to open it we found it neceſſary to cut through the maſſeter muſcles and the tendons of the temporal muſcles.

In both ſubjects, upon opening the mouth, we found a little more froth along the ſides of the tongue and palate. The ſurface of the tongue, and indeed of the whole of the mouth, was white. In the man, the œſophagus was throughout in a natural ſtate, but in the woman the internal membrane of that canal was of an aſh colour, almoſt as low down as the cardia. In both ſubjects the lungs, particularly the right lobe, were diſtended with blood, and in the woman we obſerved an ecchymoſis on the ſurface of the larger lobe, which made it appear, as it were, gangrenous.

The ſtomach of the woman was full of aliment mixed with a reddiſh frothy mucus, and ſo imperfectly digeſted, that the quality of it might be diſtinguiſhed. The man's ſtomach, on the other hand, was almoſt empty, and, inſtead of any remains of aliment, contained only about half a pint of mucus tinged with black blood and covered with froth. On removing this mucus, we diſcovered at the bottom of the

ſtomach

ſtomach a black ſpot, which, at firſt ſight, we conjectured might be the effect of gangrene; but, upon ſcraping it with the knife, we found it to be nothing more than the effect of the dark-coloured mucus adhering to the villous coat of the ſtomach, the ſubſtance of which was of its natural colour. This effect might perhaps be occaſioned by the poiſon's having remained a longer time on this than on any other part of the ſtomach.

The veſſels of the meſentery in both ſubjects, and thoſe of the ſtomach and ſmall inteſtines, particularly in the woman, were full of blood of a darker colour than uſual. The gall-bladder in the man was exceſſively diſtended with a blackiſh-coloured bile; but in the woman this receptacle was quite empty. The cauſe of this difference may perhaps be attributed to the difference in the operation of the poiſon in the ſtomachs of the two ſubjects; for in the man the ſtomach was moſt affected probably becauſe the poiſon remained in it longer than in the woman, from whoſe ſtomach it ſeemed to have paſſed ſpeedily into the inteſtines. As a farther proof of this we may obſerve, that the duodenum and half of the jejunum in the woman were inflamed, and full of a mucus mucus fimilar to that found in the ftomach of the man. In the duodenum, alfo, of the woman we difcovered fpots exhibiting an appearance like gangrene, but which, upon being fcraped, like the fpot already mentioned in the ftomach of the man, were found to be merely the effects of mucus adhering firmly to the inner coat of the inteftine.

The heart, liver, fpleen, pancreas, kidnies, and urinary bladder, in both fubjects, were found in a natural ftate, as was alfo the uterus in the woman.

We had no fooner opened the ftomach and inteftines of thefe two fubjects, than we diftinguifhed a fmell ftrongly refembling that of bitter almonds.

Such were the obfervations we made on thefe two bodies. The bottle, containing the remainder of the laurel water, was conveyed, by order of the Police, to the Town Houfe, and there, for the fake of proving its poifonous effects, fmall quantities of it were given to feveral fowls, all of which died almoft inftantly.

The frothy moifture we obferved in the mouths of thefe two unfortunate perfons confirms the remark of Strabo, that the lauro-cerafus produces a mode of death fimilar to that occaſioned by epilepſy, and ſeems to afford a freſh proof that the diſtilled water of this plant acts on the nerves.

